# Application of metagenomic next-generation sequencing in children with pneumonia of unknown etiology

**DOI:** 10.3389/fcimb.2026.1922924

**Published:** 2026-09-01

**Authors:** Haixia Huang, Yueqiang Fu, Enmei Liu, Ke Bai, Jing Li, Chengjun Liu, Yu Deng, Feng Xu

**Affiliations:** 1 Department of Intensive Care Unit Children’s Hospital of Chongqing Medical University, National Clinical Research Center for Children and Adolescents’ Health and Diseases, Ministry of Education Key Laboratory of Child Development and Disorders, Chongqing, China; 2 Chongqing Key Laboratory of Child Rare Diseases in Infection and Immunity, Chongqing, China; 3 Department of Respiratory Medicine Children’s Hospital of Chongqing Medical University, National Clinical Research Center for Children and Adolescents’ Health and Diseases, Ministry of Education Key Laboratory of Child Development and Disorders, Chongqing, China

**Keywords:** children, lower respiratory tract specimens, metagenomic next-generation sequencing, pneumonia, unknown etiology

## Abstract

**Objective:**

To investigate the pathogen spectrum and clinical application value of metagenomic next-generation sequencing (mNGS) in lower respiratory tract specimens from children with pneumonia of unknown etiology.

**Methods:**

A retrospective analysis was conducted on children hospitalized in the intensive care unit (ICU) and respiratory department ward of Children’s Hospital of Chongqing Medical University from January 2025 to December 2025. All enrolled cases presented negative results for conventional respiratory pathogen tests and received mNGS testing of lower respiratory tract specimens for etiological identification. The mNGS findings and clinical data of the included children were analyzed.

**Results:**

A total of 92 children were enrolled, including 54 males and 38 females, with ages ranging from 2 months to 13 years and 8 months. Causative pathogens were detected in 77 cases (83.7%). The clinically adjudicated etiological diagnosis rates of bacteria, viruses, fungi and atypical pathogens were 75.0% (69/92), 37.0% (34/92), 13.0% (12/92) and 5.4% (5/92), respectively. Thirty-eight cases were complicated with polymicrobial infection, among which bacterial-viral infection was predominant, accounting for 23.1% (24/92). Children with immunocompromised conditions exhibited higher incidences of clinically adjudicated bacterial, fungal and polymicrobial infection than immunocompetent patients. The most common clinically confirmed causative pathogens in immunocompromised children were Streptococcus pneumoniae, human cytomegalovirus, Haemophilus influenzae, Stenotrophomonas maltophilia and Enterococcus faecalis. Treatment regimens were adjusted in 58 cases (63.0%) based on mNGS findings, switching to pathogen-targeted anti-infective therapy.

**Conclusion:**

For pediatric pneumonia with negative conventional etiological tests, mNGS of lower respiratory tract specimens significantly enhances pathogen detection rates, effectively identifies polymicrobial infection and opportunistic pathogens. Immune status serves as a critical stratification factor influencing pathogen spectrum and infection patterns, with immunocompromised children being more susceptible to opportunistic infections. Adjustment of anti-infective regimens based on mNGS results can effectively facilitate personalized anti-infective therapy.

## Introduction

Pneumonia is a common respiratory disease in children and one of the leading causes of morbidity and mortality from infectious diseases among children under five years old worldwide, accounting for 15% of all deaths in this age group (Rahman et al., 2022). It can be caused by a wide variety of pathogenic microorganisms, with viruses being the most prevalent pathogens in the pediatric population. Acute respiratory infections triggered by viruses are also a major cause of pediatric hospital admission, and recurrent infections frequently occur during epidemic seasons (Jain et al., 2015). Despite continuous advances in diagnostic technologies, a large proportion of childhood pneumonia cases still fail to achieve a definite etiological diagnosis (Reyes et al., 2025). Although factors such as non-infectious etiologies or delayed specimen collection may contribute, it is widely hypothesized that undetected pathogens remain latent within these clinical samples. Currently, large-sample and systematic analyses of pathogen profiles based on lower respiratory tract metagenomic next-generation sequencing (mNGS) for children with pneumonia of unknown etiology remain scarce, especially studies stratified by host immune status. This study employed mNGS on lower respiratory tract specimens from pediatric patients with pneumonia of unknown etiology who had negative results on conventional pathogen testing, aiming to identify causative or potentially pathogenic microorganisms, elucidate the etiological characteristics of such cases, and retrospectively analyze clinical data to evaluate the clinical utility of mNGS in guiding diagnosis and management of children with pneumonia of unknown etiology.

## Materials and methods

### Patients

The data were retrospectively analyzed from patients hospitalized with pneumonia in the Respiratory Department and Intensive Care Unit of Children’s Hospital of Chongqing Medical University between January 2025 and December 2025. Patients meeting the following criteria were included in the study: (1) Hospitalized children aged 1 month to 14 years old; (2) Definite diagnosis of pneumonia; (3) Patients who completed both conventional microbiological tests (CMTs) and mNGS of lower respiratory tract specimens; (4) Patients with negative results of CMTs, including tests for common viruses (respiratory syncytial virus, rhinovirus, adenovirus, influenza virus, parainfluenza virus and coronavirus), common bacteria (Streptococcus pneumoniae, Haemophilus influenzae, Staphylococcus aureus and Klebsiella pneumoniae), Mycoplasma pneumoniae and Chlamydia pneumoniae. The exclusion criteria were as follows: (1) Specimens that failed to meet the quality control standards for mNGS testing; (2) Cases with incomplete clinical or laboratory data. The study was performed in accordance with the Declaration of Helsinki and was approved by the ethics committee of the Children’s Hospital of Chongqing Medical University (approval ID: 2022–498).

### Sample collection and pathogen detection

#### Conventional microbiological tests (CMTs)

Respiratory samples were collected according to standard protocols after admission. Common pathogenic bacteria were detected via smear staining, bacterial culture and mass spectrometry identification. Real-time fluorescent quantitative reverse transcription polymerase chain reaction (RT-PCR) and colloidal gold antigen assay were used to screen common respiratory viruses. Nucleic acid detection combined with serological methods was applied to identify Mycoplasma pneumoniae and Chlamydia pneumoniae.

#### mNGS testing

For bronchoalveolar lavage fluid (BALF) specimens, bronchoscopy-guided bronchoalveolar lavage was performed on the lung segments with lesions shown on chest computed tomography (CT). Normal saline was used for lavage, and the lavage fluid was collected by negative pressure suction and immediately sent for pathogen detection. For sputum specimens, deep sputum was aspirated through the endotracheal tube.

Samples of 1–4 ml of sputum, bronchoalveolar lavage fluid, pleural effusion were collected from patients according to standard protocols and stored in -80 °C before DNA extraction. For sputum samples, 0.1% dithiothreitol (DTT) was used for liquefying at room temperature for 30min. BALF and pleural effusion could go directly to the DNA extraction procedures. To begin with DNA extraction, 0.5 ml of the samples was transferred to a new 1.5ml microcentrifuge tube, DNA and RNA was extracted using the VAMNE Magnetic Pathogen DNA/RNA Kit (RM601-01, Vazyme International LLC, Nanjing, China) according to the manufacturer’s recommendation.

The extracted DNA and RNA was quantified before library preparation. DNA libraries were constructed using UltraClean Rapid DNA Library Prep Kit for lllumina (UND811-02, Vazyme International LLC, Nanjing, China). The extracted RNA performed reverse transcription reaction by UltraClean ds-cDNA Synthesis Module with gDNA wiper (UNR201-03, Vazyme International LLC, Nanjing, China), and then RNA libraries were constructed using the kit UND811–02 described above. The quality of the DNA and RNA libraries was assessed using an Agilent 2100 Bioanalyzer (Agilent Technologies, Santa Clara, California) combined with qPCR based on Applied Biosystems 7500 Real-Time PCR System (Thermo Fisher, USA), the primers based on the sequences of the adapters were used for the qPCR. Then quantified DNA and RNA libraries were pooled and analyzed on NextSeq CN500 (Berrygenomics, Beijing, China) sequencing platform. The whole process requires 48~72 hours from nucleic acid extraction to issuing the reports.

Raw sequencing reads were first quality-filtered to discard fragments shorter than 50 bp, low-quality and low-complexity sequences. High-quality reads were aligned to the human GRCh38 reference genome via Bowtie2 to remove host human sequences. Residual non-human reads were parallelly aligned to viral, bacterial, fungal and parasitic databases for microbial taxonomic annotation. All pathogenic genomic sequences were downloaded from the GenBank databases (release 259, https://ftp.ncbi.nlm.nih.gov/genomes/genbank/). The results include a list of microorganism, uniqe reads count and genome level coverage statistics. To identity suspected pathogen, we used pathogen uniqe reads per 10 million sequence (RPTM) for evaluation. Species detected in the negative control with RPTM values higher than those in the sample were removed. In addition, the following criteria should also be met: for bacterial fungal and parasite, RPTM value ≥5 was considered as positive, viral RPTM positivity threshold ≥3. For Mycobacterium tuberculosis, NTM, Nocardia, Legionella and Cryptococcus, RPTM value ≥1 was considered as positive. Species detected compared against hospital background microbial database and healthy population microbial database, the matched results were assessed as non-pathogenic and exclude from this analysis.

### Clinical diagnosis of pathogens

At present, there is no unified standard for the interpretation of mNGS results under non-sterile respiratory conditions. Based on the mNGS results, the following criteria was adopted for pathogen identification in this study (Chinese Thoracic Society, 2023; Wang et al., 2024). (1) The quality of the report and the credibility of the results were analyzed. It was determined whether the test results were standardized and credible according to the specimen type, report form, and content of the report. (2) The microorganisms detected by mNGS were classified as pathogenic microorganisms, conditional pathogenic microorganisms, or colonized microorganisms. Pathogenic microorganisms have a low potential to colonize the lung, and positive results are considered the causative agent of infection. Conditional pathogenic microorganisms should be further analyzed for host factors, blood physical and chemical indices, imaging characteristics, history of anti-infective drug use, treatment response, and traditional microbiological test results. Colonized microorganisms have a low risk of pneumonia and are generally not considered pathogenic. However, oral colonizing microorganisms may cause diseases in mixed infections such as aspiration pneumonia, lung abscess, and empyema.

To ensure the reliability of pathogen diagnostic assessment, two senior experienced clinicians independently reviewed and comprehensively interpreted epidemiological data, clinical manifestations, treatment outcomes, laboratory findings, chest imaging results and host immune status. If the two clinicians reached inconsistent judgments, a thorough discussion with an additional senior expert, or even a multidisciplinary team (MDT) consultation, was organized to achieve a unified diagnostic conclusion.

### Statistical analysis

Statistical analyses were performed using R 4.6.0 with the dplyr, flextable, and officer packages. Non-normally distributed age data were described as median (P25, P75). Categorical variables including gender, age stratification, clinical symptoms, imaging features, clinical outcomes, pathogen detection rate and clinical true positive rate were presented as percentages or constituent ratios. For intergroup comparisons, Yates-corrected Pearson’s chi-square test was adopted when the minimum theoretical frequency ≥ 5; otherwise, Fisher’s exact test was applied. A P value < 0.05 was considered statistically significant. The Benjamini–Hochberg FDR method was used to adjust P-values for multiple comparisons, and an adjusted q-value < 0.05 was defined as statistically significant.

## Results

### Characteristics of enrolled patients

A total of 92 children were enrolled, including 54 males (58.7%) and 38 females (41.3%). The median age was 3.58 years (range: 2 months to 13 years and 8 months), with two infants aged under 3 months. The clinical symptoms and imaging manifestations of all enrolled subjects are summarized in **Table 1**.

**Table 1:** Baseline characteristics of 92 pediatric patients.

Characteristics	Samples, n (%)
Sex	
Male	54 (58.7)
Female	38 (41.3)
Age [M (P25, P75), Year]	3.58 (0.74, 7.14)
Age distribution	
< 6 month	12 (13.0)
6month∼2year	26 (28.3)
2∼5 year	19 (20.7)
> 5 year	35 (38.0)
Types of pneumonia	
Community-acquired pneumonia	84 (91.3)
Hospital-acquired Pneumonia	8 (8.7)
Symptoms	
Cough	84 (91.3)
High fever	22 (23.9)
Wheezing	21 (22.8)
Tachypnea	35 (38.0)
Imaging manifestations	
Pulmonary consolidation	36 (39.1)
Atelectasis	18 (19.6)
Bronchiectasis	5 (5.4)
Pleural effusion	11 (12.0)
Ground-glass opacity	8 (8.7)
Severe cases	
ICU admission	28 (30.4)
Severe pneumonia	32 (34.8)
Respiratory failure	30 (32.6)
Sepsis	20 (21.7)
Septic shock	11 (12.0)
Non-invasive ventilation	14 (15.2)
Mechanical ventilation	26 (28.3)
ECMO	1 (1.1)
Underlying disease	44 (47.8)
Hospital stays (day)	8 (6, 16.75)

ICU, intensive care unit; ECMO, extracorporeal membrane oxygenation.

Forty-four children presented with one or more underlying diseases, including hematological malignancies (n=10), post-liver transplantation (n=4), congenital heart disease(n=8), tracheobronchial stenosis(n=1), tracheobronchomalacia(n=3), bronchiolitis obliterans(n=3), bronchiectasis(n=6), bronchial asthma(n=11), pulmonary hemosiderosis(n=1), refractory epilepsy(n=1), primary immunodeficiency disorders(n=4), chronic liver disease(n=1), and malnutrition(n=1).

### Results of mNGS detection

**Figure 1:**
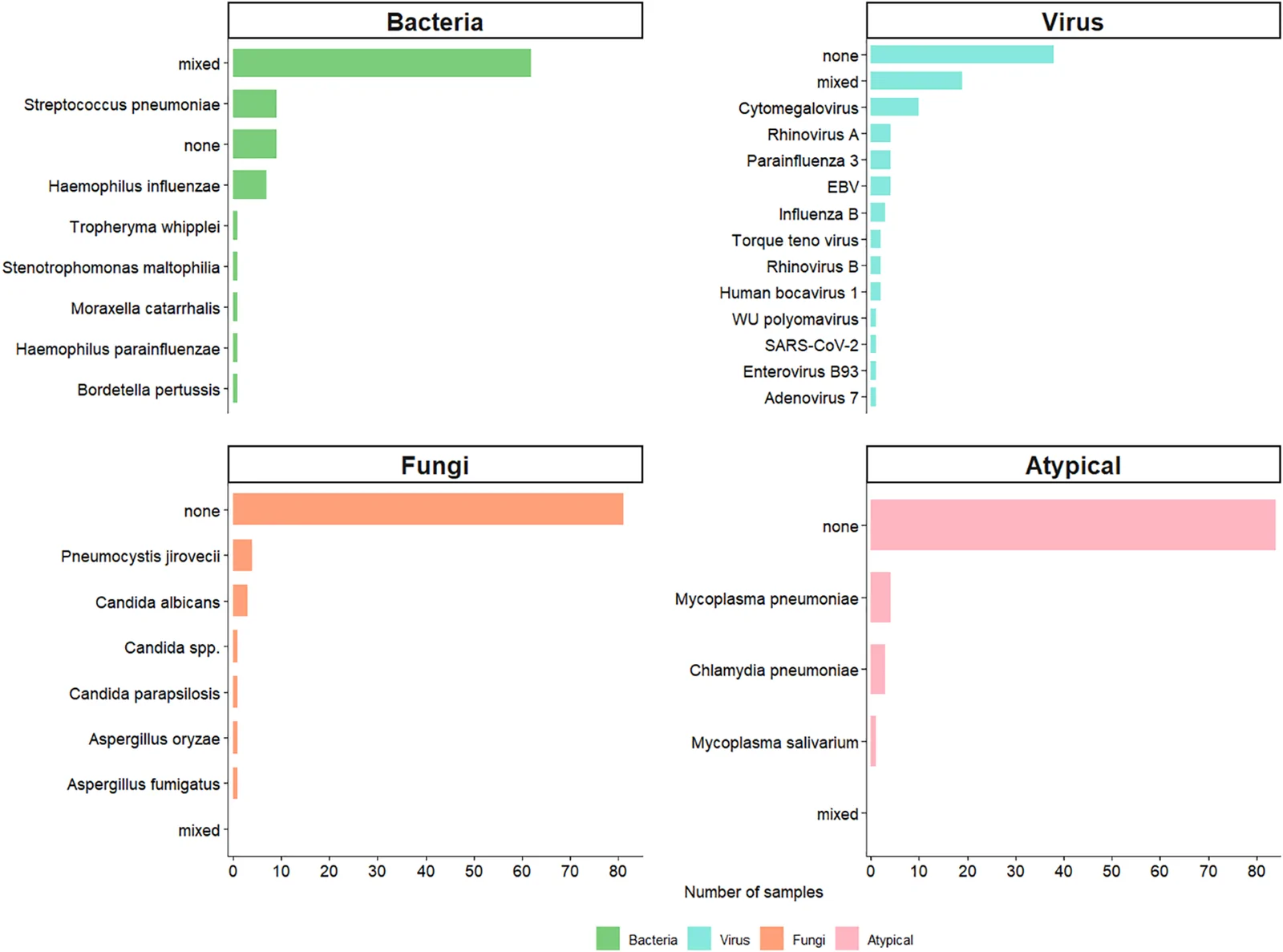
Metagenomic detections in each sample separated by microbial kingdom (n=92). EBV, Epstein, Barr virus.

All 92 lower respiratory tract samples, including 73 bronchoalveolar lavage fluids and 19 sputum samples, passed quality control. Ninety-one samples (98.9%) showed positive microbial signals via mNGS testing and only one sample (1.1%) yielded negative results with no microorganisms detected. A total of 317 microbial isolates belonging to 53 distinct microbial species were detected by mNGS among the 92 enrolled children. The mNGS detection rates for bacteria, viruses, fungi, and atypical microorganisms were 94.6% (87/92), 62.0% (57/92), 14.1% (13/92), and 8.7% (8/92), respectively. The distribution of all microbes detected via mNGS is presented in **Figure 1**.

In this study, among the detected bacteria, Streptococcus pneumoniae was the most common (n=57), followed by Haemophilus influenzae (n=27), Staphylococcus aureus (n=18), Haemophilus parainfluenzae (n=16), Stenotrophomonas maltophilia (n=14), and Acinetobacter baumannii (n=9). The five most frequently detected viruses were human cytomegalovirus (n=22), Epstein-Barr virus (n=13), rhinovirus (n=8), parainfluenza virus (n=8), and torque teno virus (n=8). Fungal microorganisms detected included Pneumocystis jirovecii (n=5), Candida albicans (n=4), Candida parapsilosis (n=2), Talaromyces marneffei (n=1), Aspergillus fumigatus (n=1), and Aspergillus oryzae (n=1). Additionally, three atypical microorganisms were detected: Mycoplasma pneumoniae (n=4), Chlamydia pneumoniae (n=3), and Mycoplasma salivarium (n=1).

### Pathogen diagnosis and distribution

Following comprehensive clinical adjudication, bacteria were verified as causative pathogens in 69 of the 87 mNGS bacteria-positive cases. Among the 57 patients with positive mNGS viral detection results, viruses were clinically adjudicated as etiological agents in 34 cases (59.6%). For the 13 patients with mNGS-detected fungi, fungal species were confirmed as infectious pathogens responsible for clinical infection in 12 patients (92.3%). As for the 8 patients with positive mNGS atypical microbial detection, 5 patients (62.5%) satisfied the clinical diagnostic criteria and were validated as the causative infectious agents of pneumonia (**Table 2**).

**Table 2:** Performance characteristics of lower respiratory tract samples results on a per-sample basis and separated by microbial kingdom.

Microbial category	mNGS detection rate (mNGS-positive cases/total cases)	Clinically adjudicated etiological diagnosis rate (causative pathogen cases/total cases)	Proportion considered clinically pathogenic (causative pathogen cases/mNGS-positive cases)
Bacteria	94.6% (87/92)	75.0% (69/92)	79.3% (69/87)
Viruses	62.0% (57/92)	37.0% (34/92)	59.6% (34/57)
Fungi	14.1% (13/92)	13.0% (12/92)	92.3% (12/13)
Atypical Pathogens	8.7% (8/92)	5.4% (5/92)	62.5% (5/8)

mNGS detection rate: Percentage of patients with positive microbial detected by mNGS, denominator = total 92 enrolled patients;.

Clinically adjudicated etiological diagnosis rate: Percentage of patients with clinically confirmed causative pathogens responsible for infection, denominator = total 92 enrolled patients;.

Proportion considered clinically pathogenic: Ratio of clinically validated causative pathogens to all mNGS-positive reads/signals.

**Figure 2:**
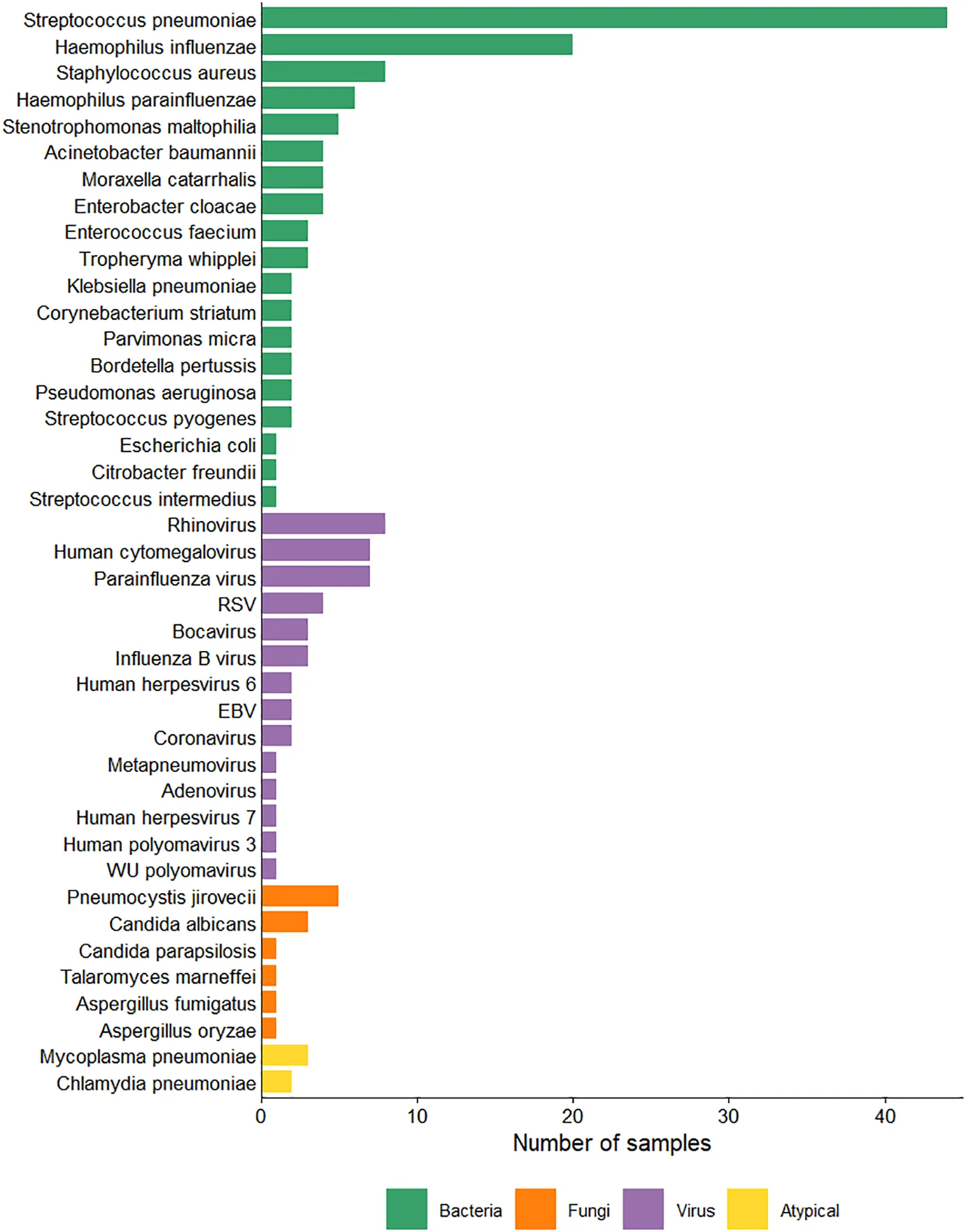
Pathogens identified in 92 pediatric pneumonia with negative conventional pathogen tests. EBV, Epstein–Barr virus; RSV, Respiratory syncytial virus.

Among the 92 enrolled patients, clinically adjudicated definitive etiological pathogens was identified in 77 cases (83.7%). The predominant bacterial pathogens were Streptococcus pneumoniae (n=44), Haemophilus influenzae (n=20), and Staphylococcus aureus (n=8). The three most common viral pathogens were rhinovirus (n=8), human cytomegalovirus (HCMV; n=7), and parainfluenza virus (n=7). Fungal pathogens primarily comprised Pneumocystis jirovecii (n=5) and Candida albicans (n=3). Two atypical pathogens were identified: Mycoplasma pneumoniae (n=3) and Chlamydia pneumoniae (n=2). Additionally, mNGS results for children had infection control and public health implications, which included coronaviruses, Bordetella pertussis, influenza viruses, and adenoviruses. The overall distribution of clinically adjudicated pathogens is displayed in **Figure 2**.

**Figure 3:**
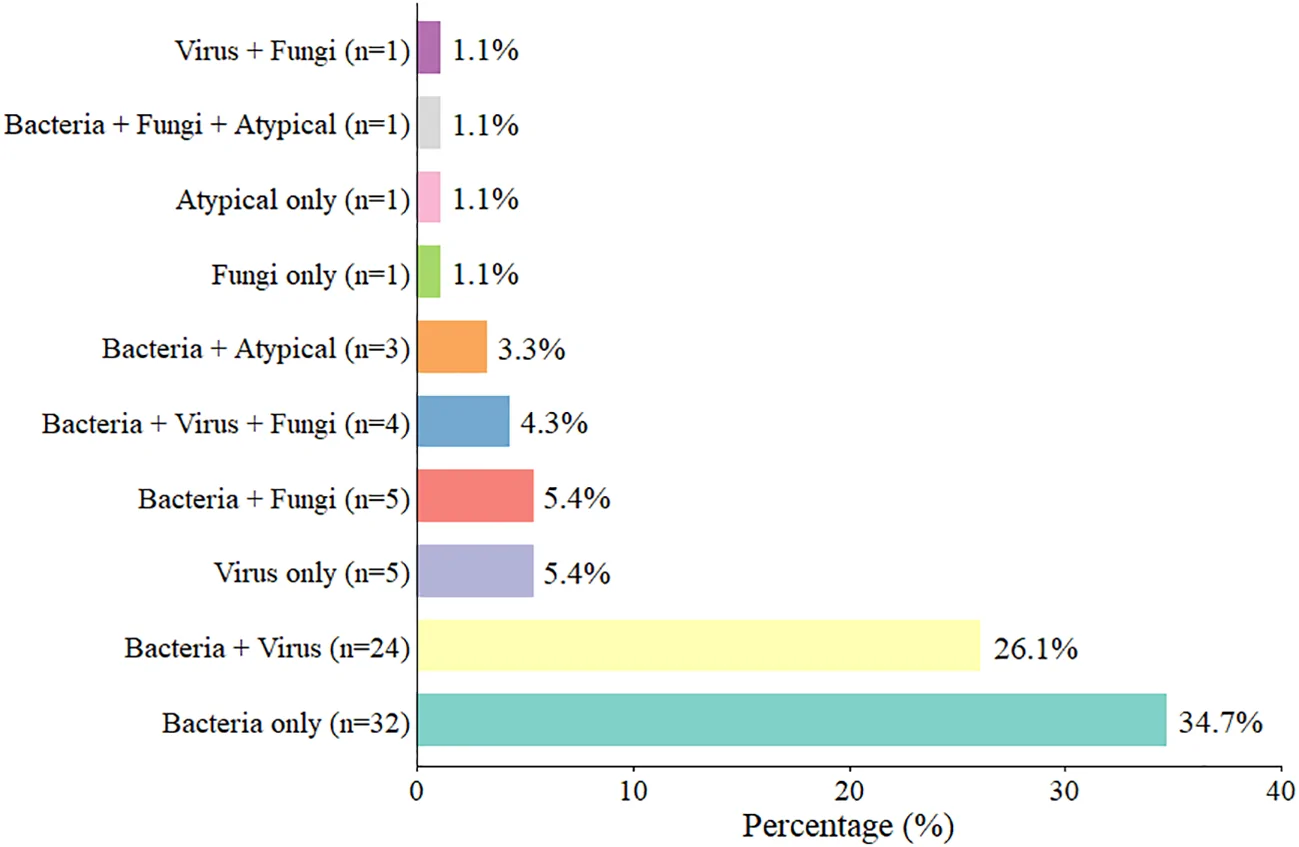
Distribution of pathogenic infection types.

In this study, infection caused by clinically adjudicated a single type of pathogen was defined as mono-infection, while infection involving two or more clinically confirmed causative pathogen types was defined as polymicrobial infection. Among mono-infections (n=39), 32 cases of bacterial infections predominated, followed by 5 cases of viral infections, with one case each of fungal and atypical pathogen infections. For polymicrobial infections, bacterial-viral infection was the most common type, accounting for 24 cases. Other combinations included 5 cases of bacterial-fungal infection, 3 cases of bacterial-atypical pathogen infection, 4 cases of bacterial-viral-fungal infection, 1 case of bacterial-fungal-atypical pathogen infection, and 1 case of viral-fungal infection. (**Figure 3**).

### Infectious conditions stratified by immune function status

Children with primary or secondary immunodeficiency, including those diagnosed with hematological malignancies, solid tumors, post-liver transplantation status, and defined primary immunodeficiency disorders, were categorized into the immunocompromised group in this study. Among the total cohort of 92 pediatric patients, 17 were classified as immunocompromised: 6 with acute leukemia, 4 post-liver transplantation, 2 with lymphoma, and 1 case each of myeloid sarcoma, post-transplantation for aplastic anemia, chronic granulomatous disease, hyper-IgE recurrent infection syndrome, and Wiskott-Aldrich syndrome. Compared with the immunocompetent group, the immunocompromised group demonstrated significantly higher frequencies of infections caused by clinically adjudicated bacteria and fungi, as well as polymicrobial infections involving two or more causative pathogen types(P < 0.05, q <0.05; **Table 3**).

**Table 3:** Comparison of pathogens between immunocompetent and immunocompromised groups.

Pathogen type	Immunocompetent group (n=75)	Immunocompromised group (n=17)	OR (95% CI)	P value	q value (FDR)
Bacteria	68.0% (51/75)	100.0% (17/17)	0.00 (0.00–0.58)	0.005	0.008
Virus	32.0% (24/75)	58.8% (10/17)	0.33 (0.10–1.11)	0.073	0.092
Fungi	6.7% (5/75)	41.2% (7/17)	0.11 (0.02–0.47)	0.001	0.003
Atypical pathogens	6.7% (5/75)	11.8% (2/17)	0.54 (0.08–6.18)	0.609	0.609
Polymicrobial infection	30.7% (23/75)	88.2% (15/17)	0.06 (0.01–0.29)	0.001	0.001

Polymicrobial infection: infection involving two or more clinically confirmed causative pathogen types.

**Figure 4:**
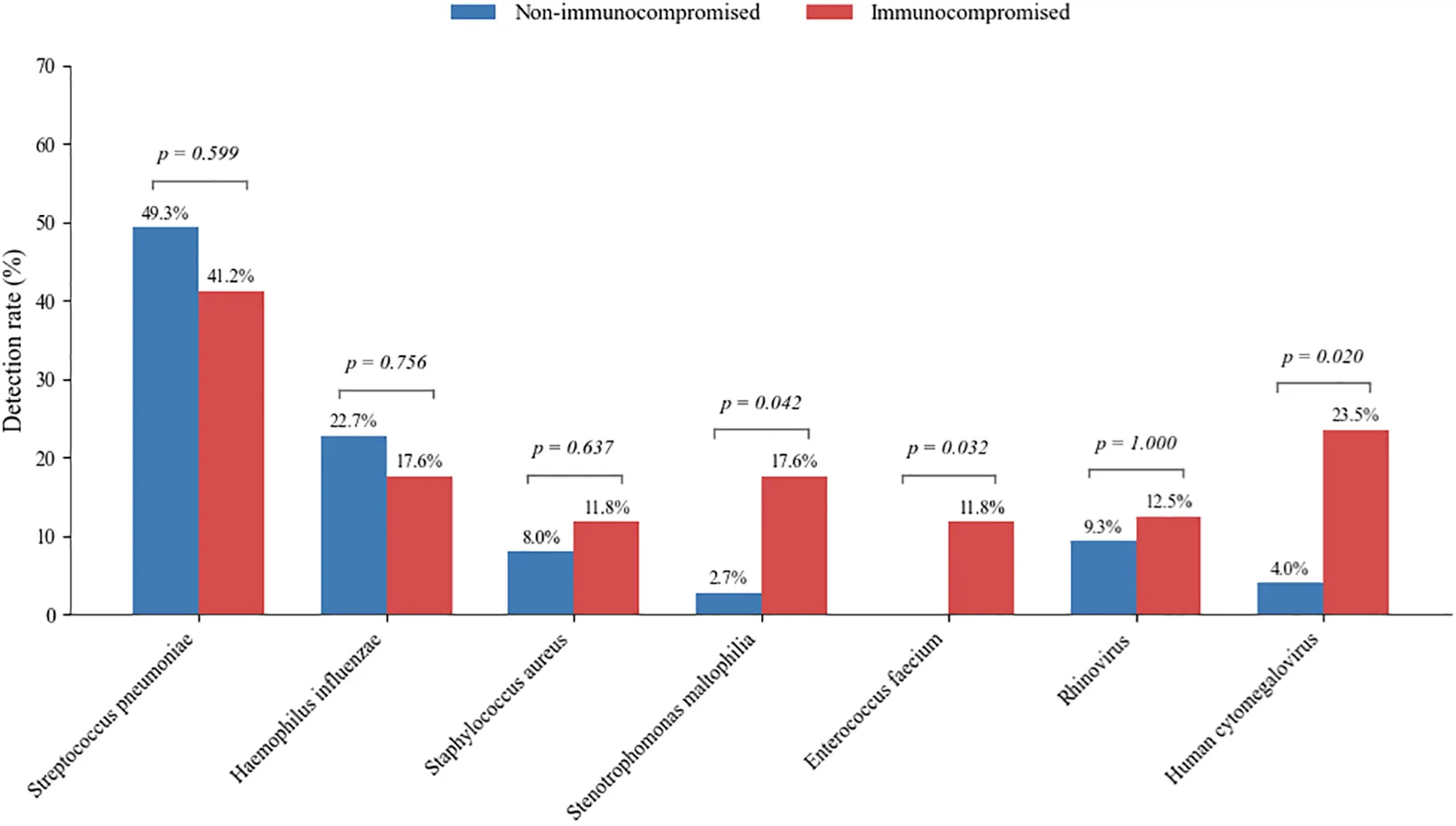
Comparison of major pathogens between immunocompromised and immunocompetent groups.

Pathogen profiling revealed that the predominant clinically adjudicated etiologic agents in immunocompromised children were Streptococcus pneumoniae, HCMV, Haemophilus influenzae, Stenotrophomonas maltophilia, and Enterococcus faecalis. In contrast, the major clinically confirmed causative pathogens infected in the immunocompetent group were Streptococcus pneumoniae, Haemophilus influenzae, rhinovirus, and Staphylococcus aureus. Notably, HCMV, Enterococcus faecalis, and Stenotrophomonas maltophilia were significantly more prevalent in the immunocompromised cohort (P < 0.05; **Figure 4**).

### Adjustment of anti-infective medication

Empirical anti-infective therapy was initiated upon admission based on the children’s medical history, clinical manifestations, chest imaging findings and relevant laboratory test results. Treatment regimens were promptly adjusted within 24 hours upon receipt of pathogen identification results. Among the 92 enrolled children, the antibiotic utilization rate prior to mNGS testing was 43.4% (40/92), and none of the patients received antiviral agents. Metagenomics results contributed to antimicrobial prescribing changes in 58 (63.0%) of 92 results. Supplementary antiviral therapy was initiated in 7 patients (7.6%), and antibiotic regimens were modified in 56 patients (60.9%). Stratified by type of antibiotic adjustment across the full cohort: 40 patients (43.5%) received additional antibiotics, 12 (13.0%) underwent antibiotic escalation, and 4 (4.3%) had antibiotic de-escalation. Complete discontinuation of antibiotics was not performed in any case.

**Figure 5:**
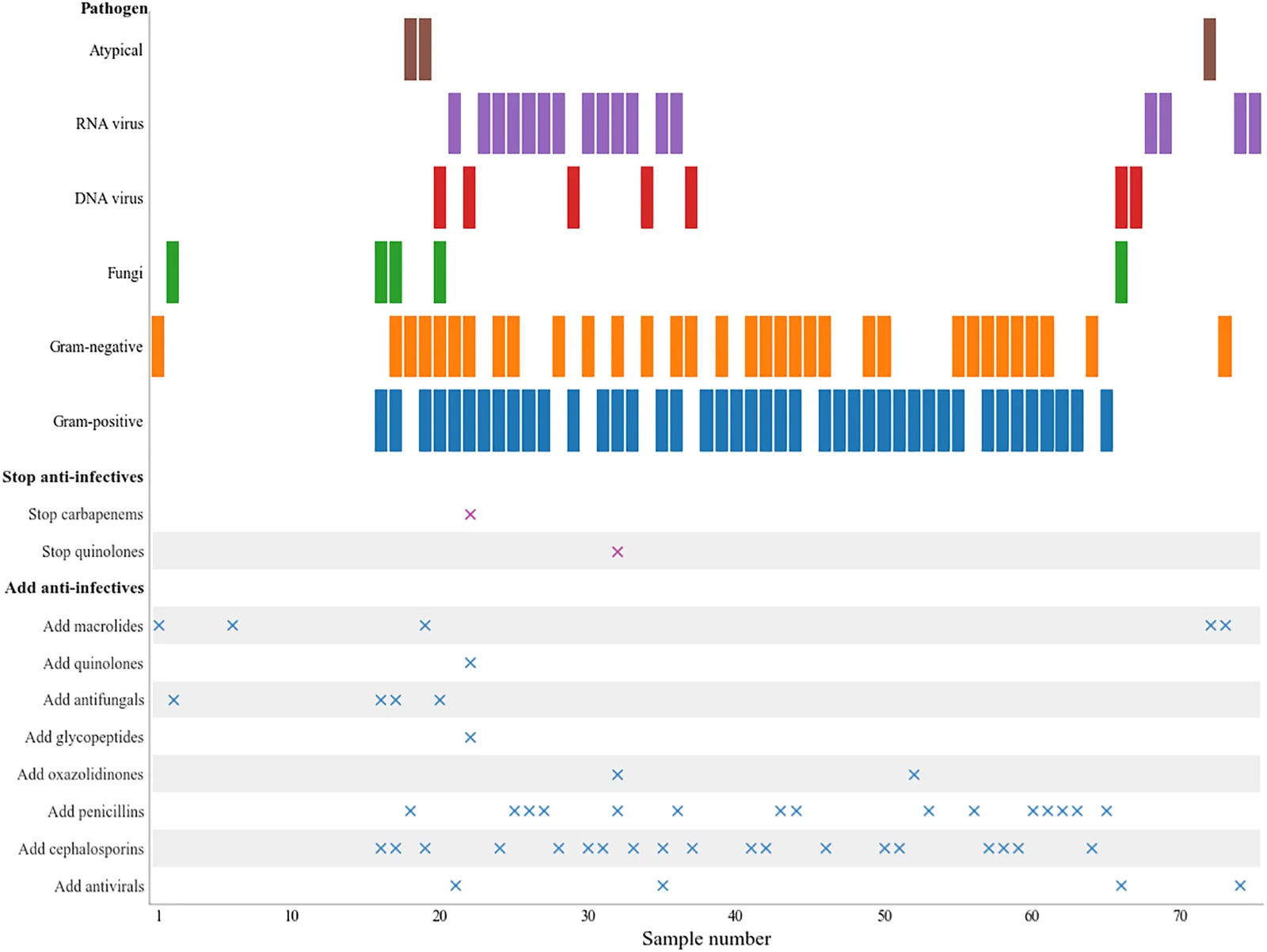
Pathogen type and antimicrobial prescribing changes attributed to metagenomics for each sample in immunocompetent group.

In the immunocompetent group (n=75), mNGS identified pathogens in 61 children (81.3%, 61/75) (**Figure 5**). Anti-infective regimens were revised for 42 of these patients (56.0%, 42/75). 41 recovered and were discharged, while 1 child with comorbid congenital heart disease and obliterative bronchiolitis developed sepsis and septic shock and was withdrawn from active treatment. Among the remaining 18 pathogen-positive cases (24.0%, 18/75) whose initial regimens already covered the mNGS-identified pathogens, no regimen changes were made; 17 recovered and were discharged, whereas 1 child with drug hypersensitivity syndrome died. One additional pathogen-positive case (1.3%, 1/75) did not undergo regimen adjustment due to pending mNGS results at discharge; despite extracorporeal membrane oxygenation (ECMO) and mechanical ventilation, clinical deterioration occurred, and care was withdrawn following family decision. Fourteen children (18.7%, 14/75) with negative mNGS results received no anti-infective modifications and were discharged after clinical recovery.

**Figure 6:**
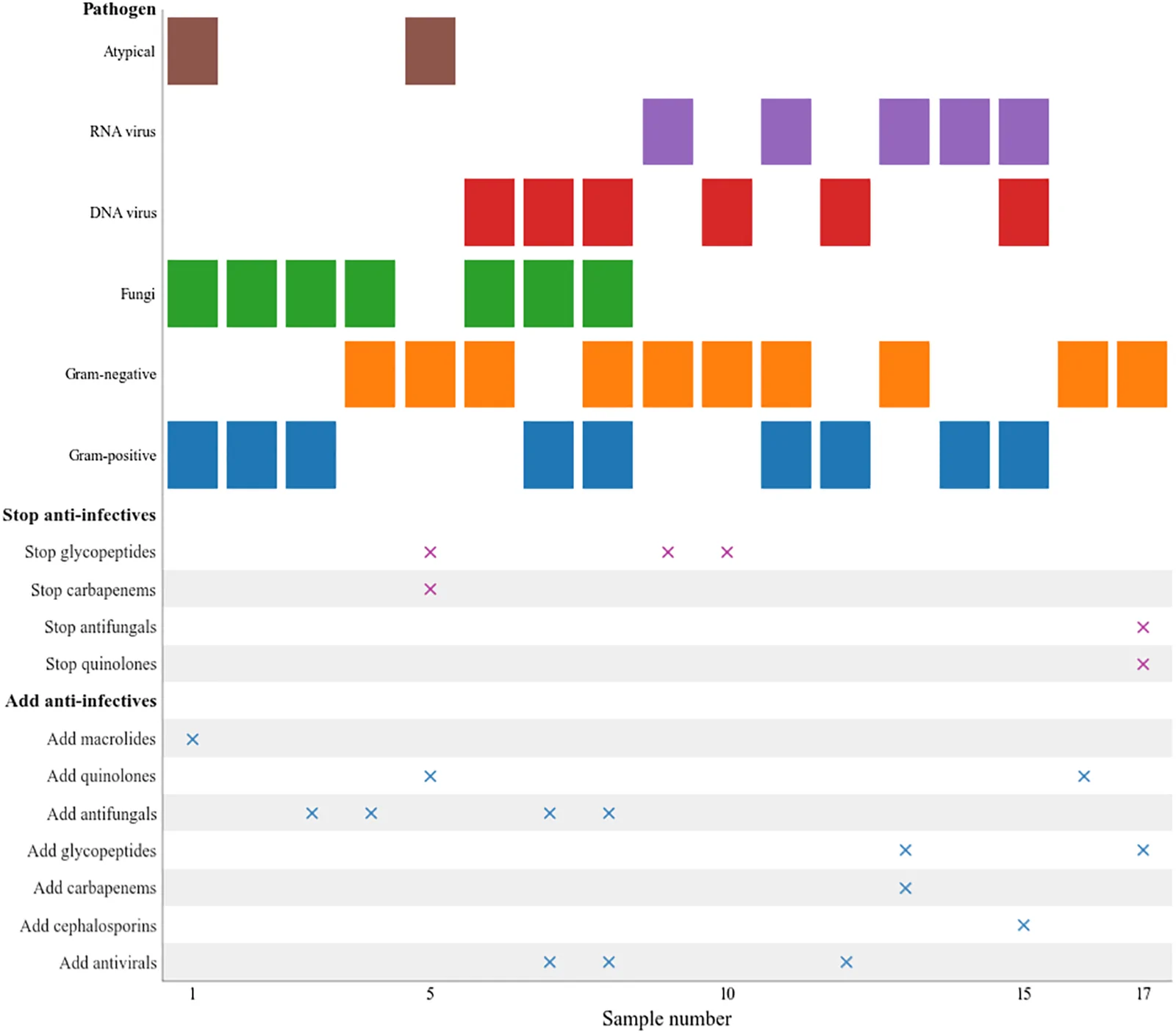
Pathogen type and antimicrobial prescribing changes attributed to metagenomics for each sample in immunocompromised group.

In the immunocompromised group (n=17), pathogens were detected in all cases. Anti-infective regimens were adjusted for 13 patients (76.5%, 13/17) (**Figure 6**). Nine children (52.9%, 9/17) recovered and were discharged; one child died of severe pulmonary hemorrhage secondary to hematological malignancy; two children withdrew care and died due to complicated sepsis, septic shock and disseminated intravascular coagulation (DIC); one child discontinued treatment after developing sepsis and septic shock with refractory hypoxemia and hypotension. Among the 3 cases (17.6%, 3/17) whose initial regimens covered the identified pathogens, 2 recovered and were discharged, while 1 was withdrawn treatment due to sepsis. The remaining one child (7.7%, 1/13) had no antibiotic adjustment at discharge pending mNGS results. The patient presented with sepsis and septic shock and failed to maintain adequate oxygenation despite high ventilator settings, leading to withdrawal of treatment.

## Discussion

Pneumonia in Children remains one of the leading global causes of pediatric morbidity and mortality (Abeja et al., 2022). Timely and accurate etiological diagnosis is critical to guiding targeted anti-infective therapy and curbing antibiotic overuse (Van De Maat et al., 2025). Nevertheless, a substantial proportion of pneumonia cases fail to yield a definite pathogen via conventional microbiological assays, including microbial culture, antigen testing and PCR. Pneumonia of unknown etiology in children represents one of the most intractable clinical challenges, frequently trapping clinicians in the dilemma of blind empirical medication8. This study systematically analyzed the pathogen spectrum identified by mNGS on lower respiratory tract specimens from hospitalized children with pneumonia and negative conventional pathogen tests, and evaluated its clinical application value. Our findings demonstrated that mNGS of lower respiratory tract samples delivers high-sensitivity and high-throughput full-spectrum pathogen detection. Stratified analysis of mNGS results by immune status enables precise discrimination of true pathogenic microbes, and supports clinical diagnostic and antimicrobial management decisions. This study provides a replicable and generalizable diagnostic and therapeutic framework for standardized management of pediatric pneumonia with unknown etiology.

The diagnostic accuracy of pathogen testing is highly dependent on specimen type. Upper respiratory tract samples are prone to interference from colonizing flora, while blood specimens exhibit limited sensitivity for focal pulmonary infections; both types may produce false-negative results. In contrast, lower respiratory tract specimens are collected directly from the infectious lesion and can more faithfully reflect the pulmonary pathogen profile (Guo et al., 2022; Meyer Sauteur, 2023; Murdoch et al., 2012). A total of 92 hospitalized children with pneumonia and negative routine pathogen tests were enrolled in this study. mNGS testing of their lower respiratory tract samples achieved an extremely high microbial detection rate of 98.9%, with bacteria being the most prevalent (94.6%), followed by viruses (62.0%), fungi (14.1%), and atypical microbes (8.7%). Combined with clinical data, clinically adjudicated causative pathogens were identified in 77 cases, corresponding to a clinical etiological attribution rate of 83.7%. Previous studies have confirmed that mNGS overcomes the limitations of conventional microbial culture for fastidious pathogens, delivering comprehensive profiling of infectious agents for clinical reference (Miller and Chiu, 2021). Multiple clinical studies have successfully applied mNGS to identify pathogens in pediatric pneumonia, with detection rates markedly superior to conventional assays (56.25% vs 72.32%; 73.3% vs 97.6%) (Li et al., 2023; Yang et al., 2022). The high detection rate observed in the present study indicates that the vast majority of children with pneumonia and negative conventional tests still harbor identifiable pathogenic microbes, and mNGS combined with lower respiratory tract sampling effectively addresses the diagnostic limitations of conventional laboratory methods.

Bacteria constitute a major category of pathogen responsible for pediatric pulmonary infections, and their rapid identification plays an indispensable role in guiding antibacterial regimens and improving treatment outcomes (Donà et al., 2017). In terms of pathogen distribution in this cohort, bacteria remained the dominant clinically adjudicated causative agents, with Streptococcus pneumoniae, Haemophilus influenzae and Staphylococcus aureus showing high clinical pathogenic positivity rates, consistent with the global epidemiological profile of common pathogens in pediatric community-acquired pneumonia (CAP) (Donà et al., 2017; Jain et al., 2015; Kulkarni et al., 2022; Vestjens et al., 2024). This finding suggests that typical bacterial infections still predominate even when conventional tests return negative results, yet they are easily missed due to prior antibiotic administration, fastidious culture requirements or suboptimal specimen quality. The predominant viral pathogens included rhinovirus, human cytomegalovirus and parainfluenza virus. Fungal pathogens were mainly Pneumocystis jirovecii and Candida albicans. Notably, Talaromyces marneffei was also detected in this study; such organisms are routinely missed by conventional assays, whereas mNGS enables rapid identification and secures a critical therapeutic window for high-risk pediatric patients. In addition, mNGS successfully detected notifiable infectious pathogens requiring priority surveillance and isolation, including coronavirus, Bordetella pertussis, influenza virus and adenovirus, conferring substantial clinical and public health value for early identification, isolation precautions and precise epidemic control.

In terms of infection patterns, patients with polymicrobial infections exhibit distinct anti-infective treatment requirements and more severe clinical manifestations compared with those with monoinfections. Combination anti-infective regimens carry inherent risks of adverse effects, which necessitates early, rapid and definitive identification of causative microbes in polymicrobial cases (Cillóniz et al., 2025; Niu et al., 2025). Prior research has validated that mNGS offers superior sensitivity and a broader detection spectrum relative to conventional etiological testing (Diao et al., 2022; Wu et al., 2025). A retrospective study conducted by Wang et al. (Wang et al., 2019) involving 55 patients with pulmonary infections reported that mNGS achieved a far higher sensitivity for diagnosing polymicrobial pneumonia (97.2%) than conventional laboratory testing (13.9%) (P<0.01). Although mNGS demonstrated lower specificity (63.2%) versus conventional assays (94.7%) (P = 0.07), the data still substantiate the prominent diagnostic advantage of mNGS for polymicrobial pulmonary infections. In the present study, dual or multiple pathogens were detected in 41.3% (38/92) of patients, with bacterial-viral infection being the most common presentation. This phenomenon may be attributed to viral damage to the respiratory mucosal barrier, which predisposes patients to secondary bacterial infection (Hendaus et al., 2015). Other frequent polymicrobial patterns included bacterial-fungal infection and triple bacterial-viral-fungal infections. These observations alert clinicians that pneumonia of unknown origin refractory to standard therapy is frequently driven by multiple co-pathogens, requiring heightened clinical vigilance.

Notably, a high mNGS microbial detection rate does not equate to a high clinical adjudicated etiological diagnosis rate. This study strictly distinguished laboratory-level microbial identification via mNGS from clinically adjudicated pathogenic status. The clinical attribution proportions for bacteria, viruses, fungi and atypical microbes were 79.3%, 59.6%, 92.3% and 62.5%, respectively. Some detected microorganisms (including certain bacteria and viruses) may merely represent colonizing flora or background microbes, which require comprehensive judgment integrated with children’s clinical manifestations, imaging features, laboratory biomarkers and immune status (Chinese Thoracic Society, 2023; Zhu et al., 2023). Although this procedure increases the complexity of clinical interpretation, it highlights the core strength of mNGS: delivering comprehensive microbiome profiles. Therefore, we recommend early collection of lower respiratory tract specimens for mNGS testing in children with pneumonia of unknown etiology, as a replacement for or supplement to repeated conventional microbiological tests.

The respiratory tract harbors a complex microbial community, and numerous colonizing or opportunistic pathogens can trigger infections, especially when host immune function is compromised. This study verified that children’s immune status exerts a decisive impact on pathogen distribution and infection patterns. The clinically adjudicated etiological diagnosis rate reached 81.3% (61/75) in immunocompetent children and 100% (17/17) in immunocompromised children. mNGS markedly overcomes the limitations of traditional assays, and immunodeficient children derive greater diagnostic benefits from mNGS, with superior clinically adjudicated etiological diagnosis rates compared to immunocompetent counterparts.

Furthermore, immunocompromised children (e.g., patients with hematological malignancies or post-transplant recipients) exhibit a significantly distinct pathogen spectrum relative to immunocompetent children. Opportunistic pathogens including human cytomegalovirus (CMV), Stenotrophomonas maltophilia and Enterococcus faecalis yielded far higher clinically adjudicated etiological diagnosis rate in immunocompromised patients, alongside a substantially higher proportion of polymicrobial infections (P<0.05). This finding aligns with previous literature, confirming that immunosuppression constitutes a high-risk factor for opportunistic and polymicrobial infections (Cheng et al., 2023; Zinter et al., 2019). In particular, the elevated detection rate of herpesviruses such as CMV among immunocompromised children underscores the vital role of viral reactivation in pneumonia within this population (Huang et al., 2023). Additionally, multiple studies have validated mNGS’s capacity to identify rare pathogens. Critical diagnoses that avoided misdiagnosis and delayed treatment have been achieved via mNGS in cases including microsporidiosis in renal transplant recipients, family-clustered Chlamydia psittaci pneumonia, and pneumonia secondary to Whipple’s disease (Li et al., 2024; Li et al., 2021; Liu et al., 2022). Collectively, these findings indicate that immune status serves as a fundamental prerequisite for interpreting mNGS results. Identical pathogen sequences may only signify colonization or transient infection in immunocompetent children, yet are highly likely to represent clinically adjudicated etiological agents in immunocompromised patients. Accordingly, clinicians must regard immune status as a core reference variable when reviewing mNGS reports, and adopt differentiated clinical decision thresholds for distinct pathogens.

The clinical value of any diagnostic technology ultimately lies in its influence on therapeutic decision-making. In this cohort, anti-infective regimens were adjusted for 63.0% (58/92) of children based on mNGS results. Antiviral therapy was initiated in 7.6%, additional antibacterials prescribed in 43.5%, antibiotic escalation adopted in 13%, while de-escalation implemented in 4.3%. These data demonstrate that mNGS not only identifies pathogens but also directly modifies clinical medication strategies to enable “precision antimicrobial therapy” (Van De Maat et al., 2025). By clarifying the full pathogen spectrum, mNGS reduces the empirical overuse of broad-spectrum antibiotics, lowering the risks of antimicrobial resistance and adverse drug reactions.

Furthermore, the clinical value of mNGS also lies in verifying the rationality of existing therapeutic regimens (Zheng et al., 2024). Eighteen immunocompetent children presented positive mNGS findings yet received no treatment adjustment, as their original regimens already covered the detected pathogens. Seventeen of these patients were discharged uneventfully, which indirectly verified the reliability of mNGS results and prevented unnecessary therapeutic modifications. Meanwhile, 14 children with negative mNGS results were discharged with clinical improvement without any treatment adjustment, indicating that negative mNGS results also serve as a valuable reference for excluding active infection and averting overtreatment.

All 17 children in the immunocompromised group obtained clinically adjudicated etiological pathogen via mNGS, and therapeutic regimens were revised for 13 patients (76.5%). Although several children suffered poor prognoses due to severe underlying disorders, mNGS provided clear etiological clues and eliminated the blindness of empirical therapy. Two patients in this study failed to receive timely regimen adjustments owing to delayed mNGS reports and eventually experienced clinical deterioration, highlighting the critical impact of test turnaround time on clinical decision-making for critically ill patients. Apart from the persistent challenge of a lengthy workflow spanning data acquisition, bioinformatic analysis and report issuance that restricts real-time clinical utilization of current mNGS protocols (Zhang et al., 2022), another key principle should be emphasized: for critically ill and immunocompromised children, mNGS testing should be ordered simultaneously with conventional microbiological assays rather than reserved as a second-line diagnostic tool (Tsitsiklis et al., 2022).

This study has several limitations. It is a single-center retrospective investigation with a limited sample size. In-depth correlation analyses between pathogen load, read counts and disease severity were not performed, and no rigorous control group was established to compare prognostic differences between mNGS and conventional testing. Future research may expand the sample size and launch multicenter prospective trials to further explore correlations between mNGS pathogen read counts, pathogen species, and the severity, therapeutic response and long-term prognosis of childhood pneumonia. Additionally, interpretative criteria for mNGS outputs should be optimized to distinguish pathogenic microbes from colonizing flora, so as to further enhance the clinical guiding value of this assay.

In conclusion, for pediatric pneumonia with negative conventional pathogen tests, early mNGS testing of lower respiratory tract specimens enables efficient identification of causative pathogens, polymicrobial infections and rare covert pathogens. Stratified interpretation of mNGS results should be performed based on the child’s immune status. For immunocompetent children, attention should be focused on common community-acquired pathogens; for immunocompromised children, high vigilance is required against opportunistic pathogens (including HCMV, Pneumocystis jirovecii, Stenotrophomonas maltophilia) and polymicrobial infections. Targeted anti-infective regimens can be precisely adjusted following stratified interpretation, including adding pathogen-specific agents, simplifying existing medication regimens, or confirming the appropriateness of ongoing treatment. This approach reduces blind empirical medication, provides an important reference for standardized diagnosis and management of difficult-to-treat pediatric pneumonia, and facilitates more efficient clinical evaluation and treatment for such cases.

## Data Availability

The raw data supporting the conclusions of this article will be made available by the authors, without undue reservation.
